# N-Tools-Browser: Web-Based Visualization of Electrocorticography Data for Epilepsy Surgery

**DOI:** 10.3389/fbinf.2022.857577

**Published:** 2022-04-21

**Authors:** Jay Burkhardt, Aaryaman Sharma, Jack Tan, Loraine Franke, Jahnavi Leburu, Jay Jeschke, Sasha Devore, Daniel Friedman, Jingyun Chen, Daniel Haehn

**Affiliations:** ^1^ Machine Psychology Lab, Department of Computer Science, University of Massachusetts Boston, Boston, MA, United States; ^2^ Department of Neurology, New York University, Grossman School of Medicine, New York, NY, United States

**Keywords:** epilepsy, electrode, ECOG, visualization, electrocorticography, seizure, surgery, webgl

## Abstract

Epilepsy affects more than three million people in the United States. In approximately one-third of this population, anti-seizure medications do not control seizures. Many patients pursue surgical treatment that can include a procedure involving the implantation of electrodes for intracranial monitoring of seizure activity. For these cases, accurate mapping of the implanted electrodes on a patient’s brain is crucial in planning the ultimate surgical treatment. Traditionally, electrode mapping results are presented in static figures that do not allow for dynamic interactions and visualizations. In collaboration with a clinical research team at a Level 4 Epilepsy Center, we developed N-Tools-Browser, a web-based software using WebGL and the X-Toolkit (XTK), to help clinicians interactively visualize the location and functional properties of implanted intracranial electrodes in 3D. Our software allows the user to visualize the seizure focus location accurately and simultaneously display functional characteristics (e.g., results from electrical stimulation mapping). Different visualization modes enable the analysis of multiple electrode groups or individual anatomical locations. We deployed a prototype of N-Tools-Browser for our collaborators at the New York University Grossman School of Medicine Comprehensive Epilepsy Center. Then, we evaluated its usefulness with domain experts on clinical cases.

## 1 Introduction

In the United States, around one million people have epilepsy that cannot be controlled with anti-seizure medications (Engel, 2016; Zack and Kobau, 2017). Surgical intervention is often necessary for these patients and becomes the main path to treatment. Some surgical epilepsy patients undergo a two-stage surgical procedure during which 1) electrodes are implanted in the brain to help clinicians identify the location(s) of the seizure focus followed by 2) the resection or ablation of the pathological tissue. During the first stage of the surgery, electrical activity across broad brain regions is recorded through the implanted surface with depth electrodes. The seizure focus location is estimated based on patterns of abnormal brain activity during seizures. It is used to determine the resection or ablation boundaries in the second stage. Before resection, electrical stimulation mapping is typically performed to determine whether the identified areas are eloquent, i.e., functionally critical for language, memory, sensation, or motor responses, which would preclude them from a resective procedure. Thus, pre-surgical planning for the second stage of epilepsy surgery requires registration of epileptiform activity and functional mapping results on the patient’s brain.

**FIGURE 1:**
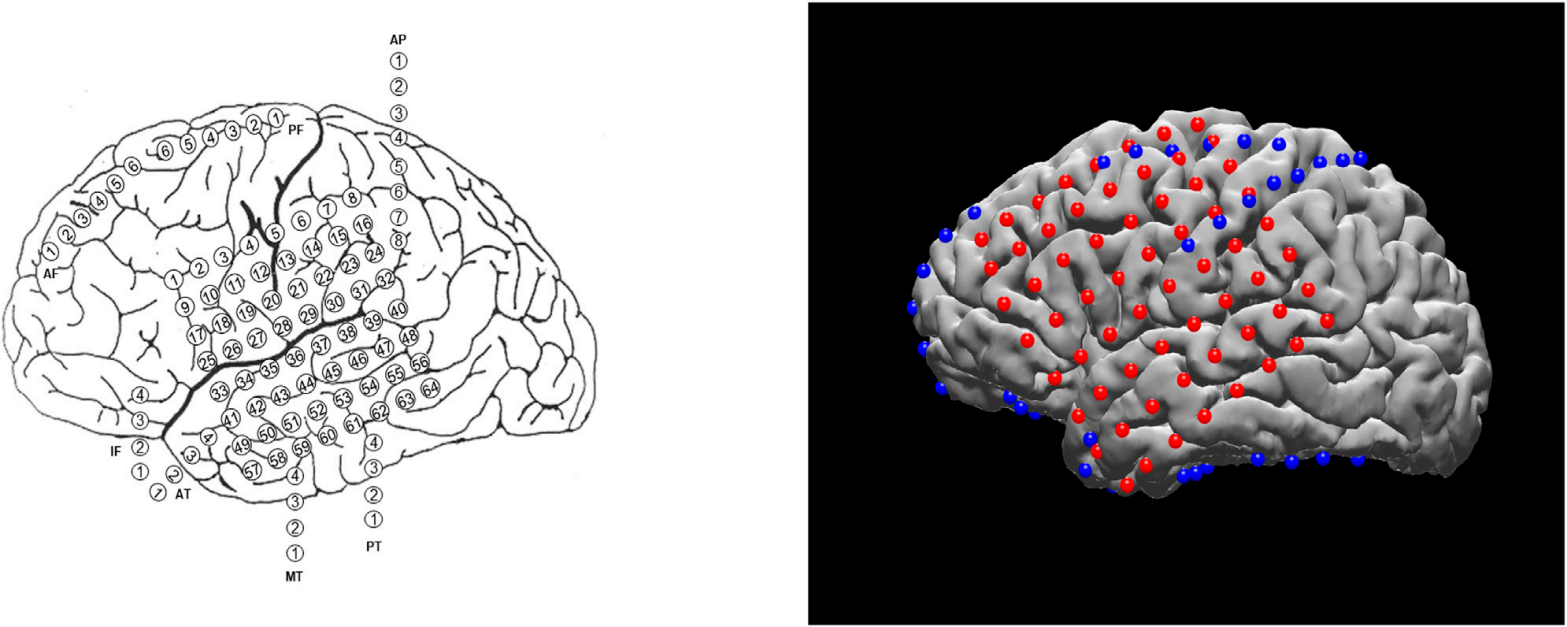
Different visualization methods for the same ECoG data set. Left: a schematic drawing. Right: a static 2D figure from N-Tools-elec.

Planning the surgical approach to the treatment of drug-resistant focal epilepsy involves the integration of multiple sources of data to determine the brain areas to target for resection in order to maximize the chance of cure while minimizing the risks of neurological deficits. In many cases, neurologists and neurosurgeons employ intracranial electrodes to precisely identify sites of seizure initiation and spread as well as identify eloquent brain areas to avoid. In clinical practice, the spatial distribution of electrodes is first recorded manually by neurosurgeons, either via hand drawings or schematic plots (see example on the left of Figure 1). Although these raw materials serve as essential references for surgical planning, they are not intended to convey the precise anatomical locations of these electrodes. The clinicians must then mentally translate this two-dimensional data onto the complex three-dimensional structure of the patient’s own brain, in order to determine if the observed data supports their localization hypothesis (based the presence of brain lesions or the anatomical-clinical manifestation of the seizure) and then plan the best surgical approach (Jobst et al., 2020). There are limited visualization approaches for this data and most require specialized software for viewing and annotation. Using post-operative Computed Tomography (CT) or magnetic resonance imaging (MRI) scans coregistered to the pre-operative MRI, the physical location of electrodes can be registered directly to patient brains. The visual results of electrode localization procedures typically contain schematics of electrodes overlaid on an individual patient’s brain (for subject-level analyses) or a template brain (for group-level analyses). The right panel of Figure 1 shows an example electrode localization that was generated by the open-source toolkit, N-Tools-Elec (Yang et al., 2012).

N-Tools-Elec is a semi-automated Matlab toolkit developed by New York University (NYU) ‘s Comprehensive Epilepsy Center (CEC). It can accurately map electrode coordinates onto a 3D brain surface reconstructed from an MRI scan and determine the type of brain tissue where the electrodes are located. The N-Tools-Elec mapping algorithms were also implemented in the open-source toolkit iELVis, by Groppe et al. at the University of Toronto (Groppe et al., 2017).

Traditionally, the visualization outputs of N-Tools-Elec are stored as static pictures, with a pre-set camera configuration without any functionality enabling the user to change the view or dynamically interact with the output. A web-based, easy to use visualization tool that can be deployed anywhere in the hospital–not only in the operating room to guide surgery but also at the patient bedside to facilitate education and counseling - would be extremely useful to clinicians. In this paper, we will introduce such a web-based visualization tool, N-Tools-Browser, that extends N-Tools-Elec and allows for real-time camera interaction in a web browser.

N-Tools-Browser is designed to be a fully interactive and easy-to-use ECoG tool that runs directly in the browser and streamlines the electrode visualization process for clinicians involved in pre-surgical planning. N-Tools-Browser maps and displays electrodes (represented as spheres) on both a 3D brain surface that is reconstructed from an individual patient’s brain scan as well as 2D brain slices in three different anatomical positions: coronal, sagittal, and axial. By selecting a specific electrode in the 3D map, the 2D images automatically update to the correct anatomical position and all the relevant attributes for that electrode are displayed in the user interface. The workflow for N-Tools-Browser is divided into three steps.

**FIGURE 2:**
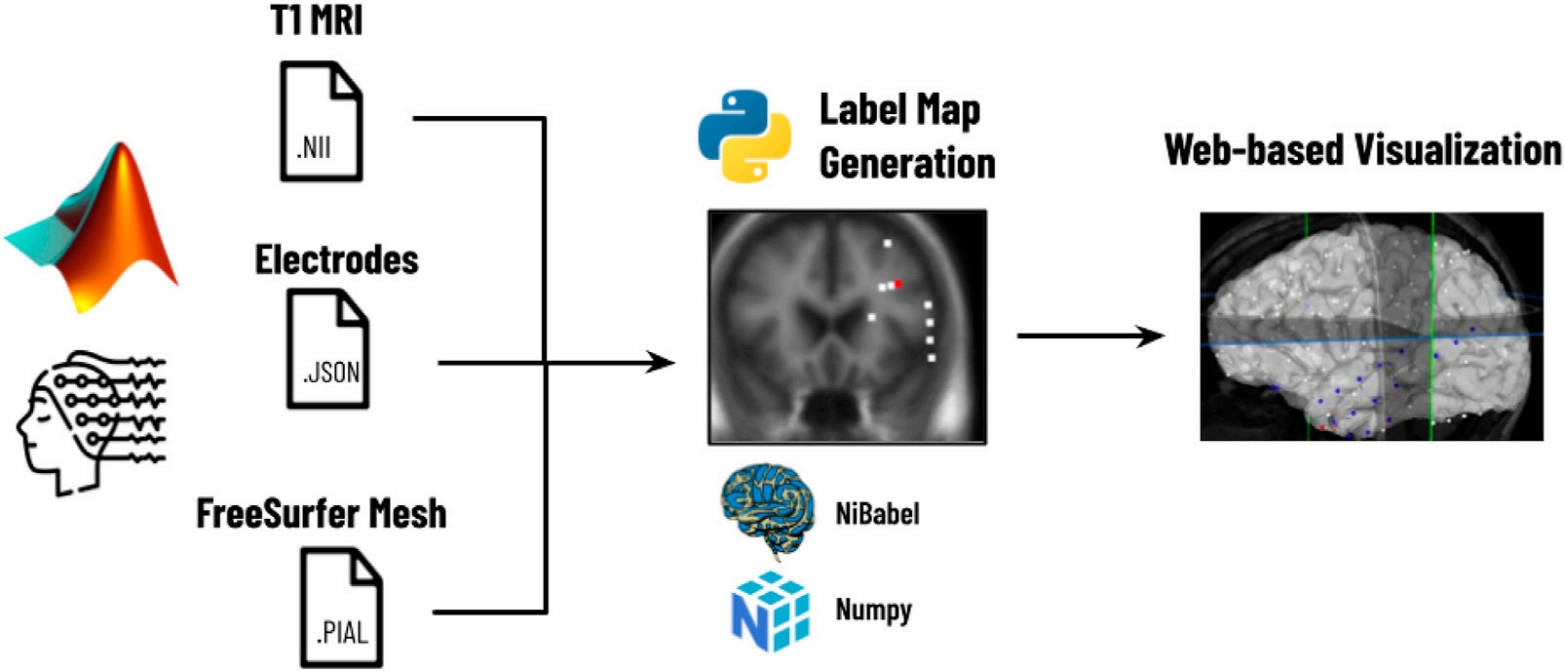
Workflow for N-Tools: In the first step, the user runs a patient’s electrode data through a MATLAB preprocessing pipeline to generate a T1 MRI NIfTI file, a JSON file, and a FreeSurfer Mesh file. In the second step, a Python script takes the patient’s JSON and NIfTI files and generates a label map of the different seizure types. Finally, the T1 MRI NIfTI file, the JSON file, and the label map are loaded into N-Tools-Browser for visualization.

As shown in Figure 2, the first step is the reconstruction, where a user runs N-Tools-Elec scripts in MATLAB and produces a brain surface mesh file (reconstructed from T1-weighted MRI via Freesurfer), and a JSON file containing electrode attributes and stimulation mapping. Second, the user runs a Python script on the patient’s JSON and NIfTI file to generate a label map for the different seizure types. Finally, the JSON, NIfTI, mesh files, and the label map are used to generate the 2D and 3D visualizations in N-Tools-Browser. We evaluated the satisfaction of clients and the impact of our tool through an expert study. Through a structured list of tasks and the NASA-TLX standard survey, two expert clinicians from the New York University School of Medicine provided us with qualitative feedback.

## 2 Related Work

Electrocorticography (ECoG) measures the electrical activity in the brain through electrodes that are placed directly on the cortical surface or in the brain parenchyma (Amaral et al., 2007). Visualization is a crucial element for processing ECoG data. While some stand-alone software tools are primarily used for visualization purposes (e.g., Visbrain (Combrisson et al., 2019)), the visualization modules are generally integrated into more comprehensive analysis packages, such as Brainstorm (Tadel et al., 2011), MNE (Gramfort et al., 2013), NeuralAct (Kubanek and Schalk, 2015), N-Tools-Elec (Yang et al., 2012), iELVis (Groppe et al., 2017), and ECoG ClusterFlow (Murugesan et al., 2017), etc. These conventional ECoG visualization software tools often require a local download and installation of a software package onto a workstation. While visualization on local computers has advantages in data safeguard and maintenance, it has limitations when multiple users, such as a team of neurosurgeons and epileptologists, need joint access.

In order to address some of these limitations of traditional software, several web-based medical imaging tools have been proposed. Biomedisa, developed at Heidelberg University, is an example of an open-source medical imaging library which has been shown to handle a wide variety of volumetric data (Lösel et al., 2020). Another online tool, GradioHub, has been proposed as a collaborative way for clinicians and biomedical researchers to share and study data (Abid et al., 2020). Finally, the open-source tool Studierfenster has been recently been created for the purpose of biomedical data visualization, enabling rendering of both 3D and 2D data, as well as letting the user annotate the data they are examining (Egger et al., 2022). These tools represent a small fraction of the many web-based medical imaging libraries available.

Recent efforts aimed to develop web-based visualization of neuroimaging data to enable real-time sharing between multiple users. For example, Mislap et al. at Johns Hopkins University (JHU) developed WebFM, a browser-based toolkit for brain-computer interfaces and functional brain mapping (Milsap et al., 2019). Also, at JHU, the Cognitive Neurophysiology and Brain-Machine Interface Lab developed an ECOG reconstruction suite (https://github.com/cronelab/ReconstructionSuite) that supports web-based visualization. Other tools such as RAVE (Magnotti et al., 2020) have been used for EEG data and some types of intracranial electrodes with a web interface.

To enable web-based interfaces, the use of open-source Javascript libraries for 3D web-based visualization of medical imaging data is often required. An emerging number of visualization techniques and software tools have been developed for neuroscientific purposes, which may be one of the largest fields in medical imaging (Franke and Haehn, 2020). For example, the framework Brainbrowser (https://brainbrowser.cbrain.mcgill.ca) is a well-established WebGL library with powerful graphical features (Sherif et al., 2015). However, it has restrictive requirements for input data, such as format and naming schemes. Compared to Brainbrowser, the A* Medical Imaging (AMI) toolkit (https://github.com/FNNDSC/ami) (Rannou et al., 2017) is more flexible and easier to deploy, but development seems stalled based on Github commit activity.

XTK was the first library for web-based medical image visualization and had a straightforward, open API (https://github.com/xtk/X) (Haehn et al., 2014). Our N-Tools-Browser (which extends the NYU N-Tools-Elec toolkit) is a novel web-based visualization tool based on XTK. N-Tools-Browser is easily accessible via a web browser and contains a responsive user interface that is inspired by SliceDrop, a web-based viewer for medical imaging data (Haehn, 2013). Users can hover over and click on electrodes in 3D space. The UI responds by displaying relevant information about the particular electrode on a panel and moving the 2D slices to the selected location.

## 3 Design Objectives

We consulted with researchers and clinicians at the NYU CEC to develop the specific requirements for our development efforts. Core to our design principles of N-Tools-Browser is a simple, straightforward, and easy-to-use graphical user interface (GUI) for clinicians to access the information they need during pre-surgical planning quickly. We want clinicians to be able to look at the attributes of each electrode, such as the electrode ID, electrode type, and seizure type, to aid them in pinpointing the seizure focus. In semi-structured interviews with domain experts from the NYU CEC, we defined the following design objectives of our tool to create a useful visualization tool.

D1) Create a 3D display of a specific patient, populating a brain mesh with sphere objects to represent electrodes on the patient’s brain and displaying colorful electrodes on 2D slices with a label map overlay.
D2) Provide functionality for switching between seizure types and functional mappings.
D3) Enable clinicians to correctly identify the anatomical region of a particular electrode along with other relevant information (ID, coordinates, interictal population, seizure type, functional mapping, etc.).

## 4 Implementation

To be consistent with our design objectives, we aimed for a simple and user-friendly implementation that facilitates the tasks of domain experts, surgeons, and clinicians.

### 4.1 Electrode Visualization on 3D Brain Surface

N-Tools-Browser uses the X-Toolkit (XTK) API(Haehn et al., 2014) for 3D visualization as it offers many built-in features for working with medical imaging data. XTK can load and parse NIfTI images and mesh surfaces and then render them in 3D space. XTK also supports basic geometric shapes, such as spheres and cylinders, which we use to represent electrodes and functional mappings, respectively. The rendering process begins by using XTK’s volume and mesh objects which can render NIfTI and mesh surface files from FreeSurfer (Fischl, 2012). The files can be stored either locally or remotely on a server. These objects are then added to four different renderers. The first renderer handles the displaying of 3D objects on the scene, whereas the remaining renderers handle the 2D slices of the NIfTI file in the saggital, horizontal, and coronal planes.

A JSON file containing a series of arrays is loaded after initializing all the renderers. The arrays all have the same length as the number of electrodes in the data set. Each array index corresponds to a different electrode, i.e., the 0th index in each array corresponds with the first electrode in the data-set. Each array also stores additional electrode attributes, such as the electrodes identification number, coordinates, group type (grid, strip, depth, etc.), associated seizure type (1, 2a, 2b, etc.), and attribute (onset, early spread, etc.), interictal population, as well as different functional mappings (language, motor, etc.). The JSON is then parsed, and each electrode is mapped to an XTK sphere object and rendered onto the scene (D1).

**FIGURE 3:**
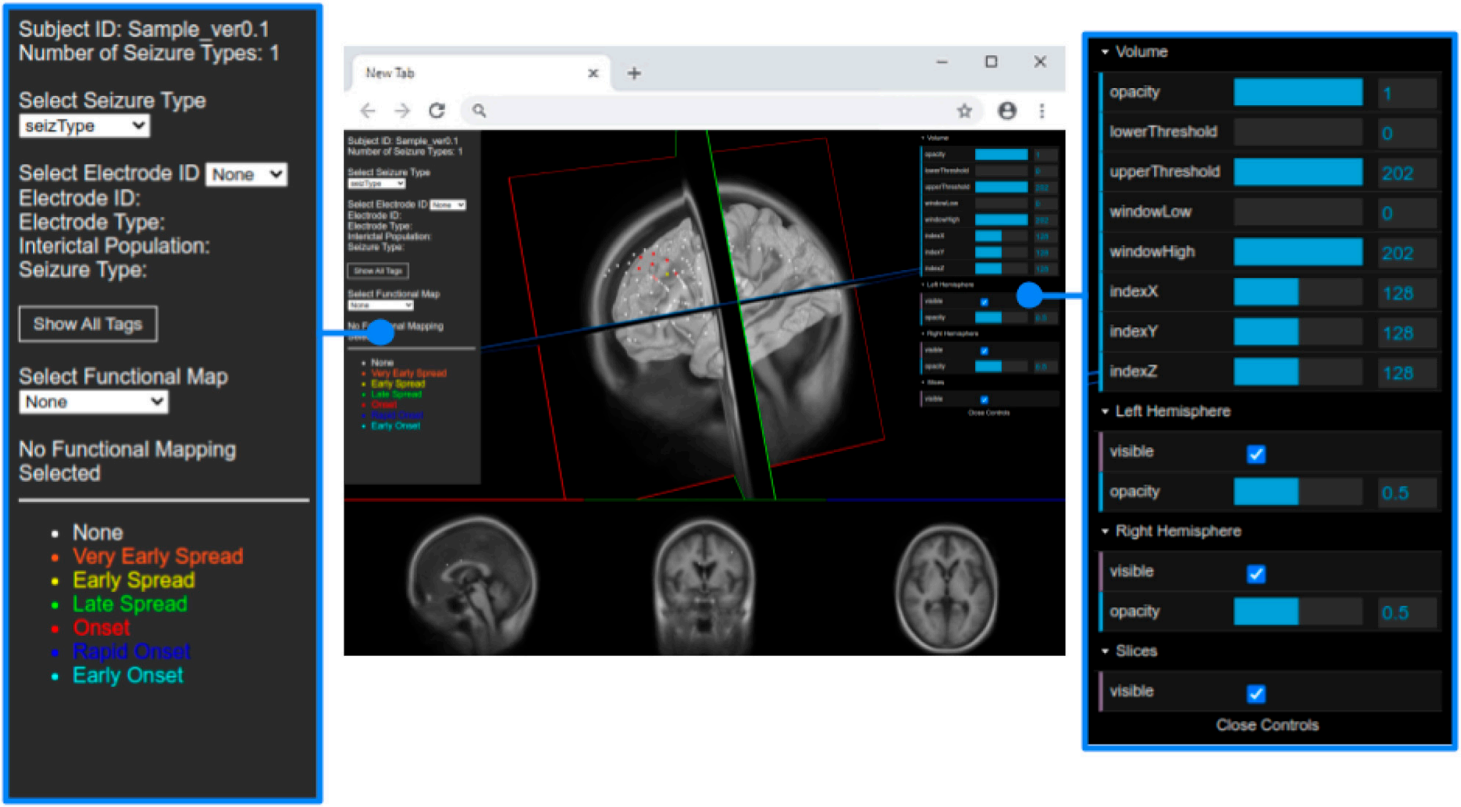
Full view of the user interface after loading the data set of a patient. Detailed view of the GUI control panel to adjust different visualization properties of the brain mesh and 2D slices.

**FIGURE 4:**
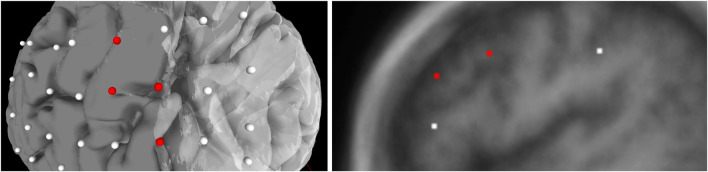
Population of electrodes on the brain in 3D space (left) and on a 2D slice (right) in the *x*-direction. The red electrodes represent the “onset” attributes of associated seizure type, while white indicates no seizure type is associated to the electrode.

The JSON file also contains an array for each seizure type of a particular subject. Each type of seizure activity is mapped onto a specific color, and a legend showing this mapping is displayed in the UI. Figure 3 provides an example of this legend for a particular seizure type, and Figure 4 shows electrodes of type “onset” mapped to a brain surface after loading.

### 4.2 Electrode Visualization on 2D Slices

To implement the two-dimensional visualization of electrodes on brain slices, we used a Python script that reads the JSON data and creates a label map for each seizure type. Using the Python script to generate label maps enables the user to switch between them more efficiently. Since the label map is essentially a static overlay to the original scan, the label maps and original scan can exist in the same renderer, eliminating the need for multiple renderers. Generating the label map data also ensures the data will be ready to use from launch, and not slowly re-generated during each rendering. Each dimension of the array represents a two-dimensional slice. The coordinates, which appear on an interval from -127.5 to 127.5, must be placed in a slice indexed from 0 to total number of slices in the data. We do this with the following equation that takes as input an input range [*a*, *b*], an output range [*c*, *d*], and a coordinate:

$s_{i}=\frac{b-a}{d-c}\left(coordinate-c\right)+a$

In this equation, *s*_*i*_ refers to the section of the MRI scan on which a 3D coordinate is mapped. In our case, the input contains the possible range for a coordinate, and the output includes the number of slices. For example, a coordinate with value -32.7 on the interval [ − 127.5, 127.5] will be matched with slice number 95 on the interval [0, 255] when rounded to the nearest integer. After calculating an index for each coordinate, we change the value of the array at that index to a predetermined constant matching a specific electrode color, and the array is saved as a NIfTI file. The volume parser in XTK already has functionality for adding label maps, so this new NIfTI file is loaded on top of the already existing file. This allows for electrodes to appear on slices without any additional rendering as they now appear as one image (D2/D3).

### 4.3 User Interface

**FIGURE 5:**
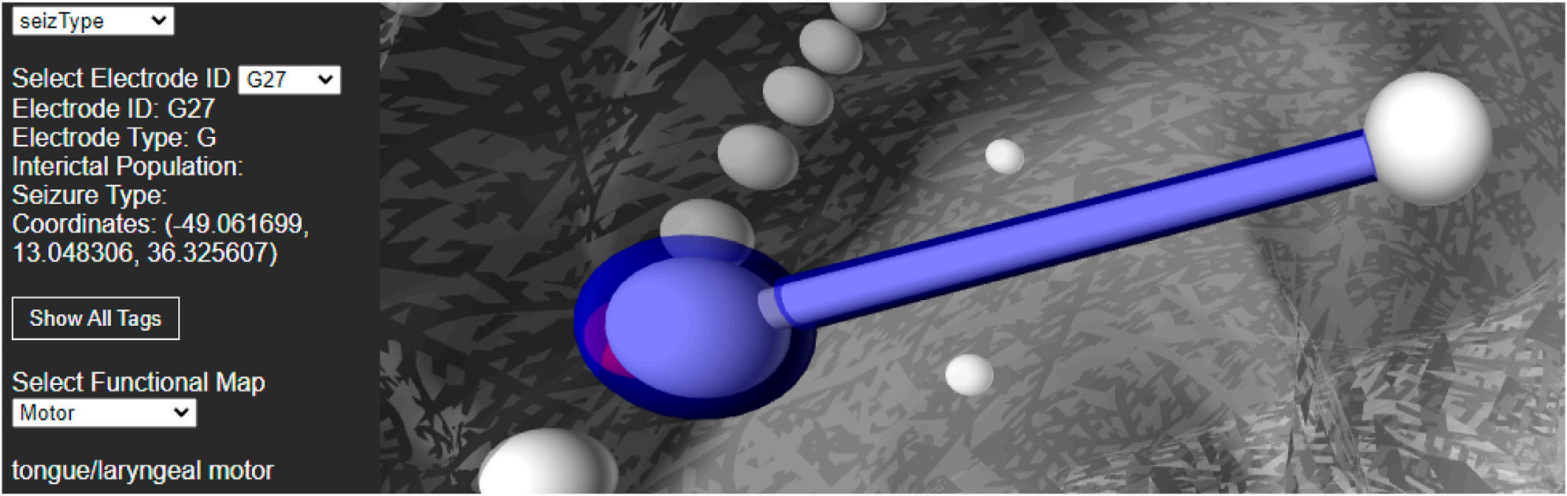
Information displayed about an electrode (G27) and functional mapping when clicked by a user. Selecting a functional map from the drop down menu will display the connected electrode pairs involved.

**FIGURE 6:**
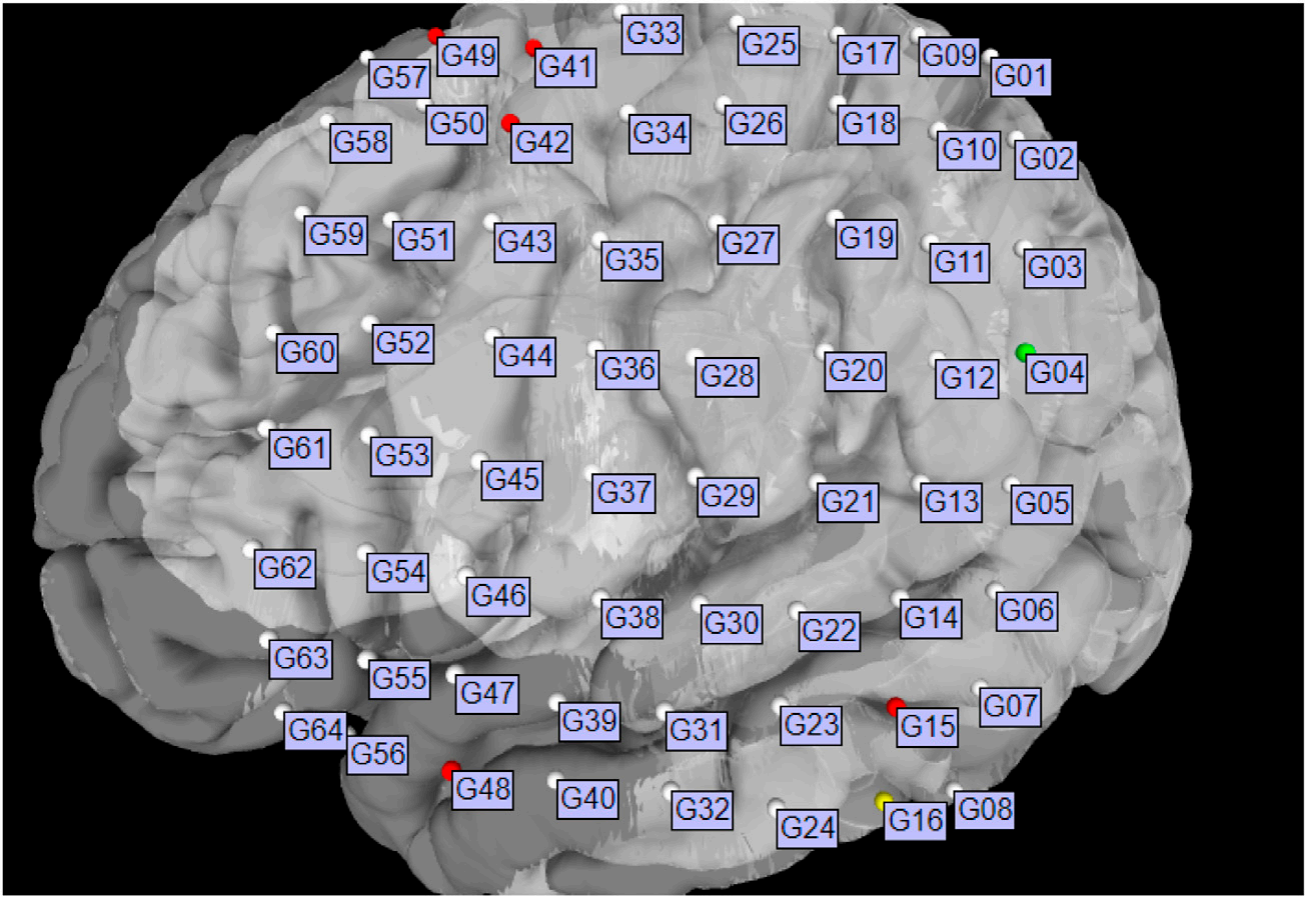
Brain surface mesh with all electrode IDs displayed simultaneously.

**FIGURE 7:**
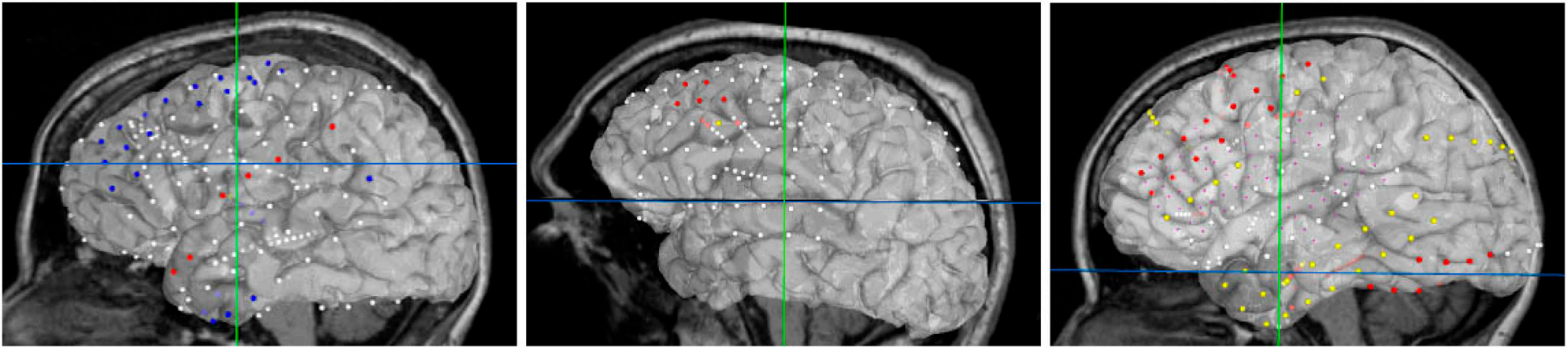
Final browser-based visualization of three different subjects NY704, NY758, NY836 including localization of electrodes on the brain surface. The color of each electrode represents the different attribute (onset, early spread, etc.) for the corresponding seizure type.

For best usability, we designed our user interface to show all the relevant information of an electrode in one panel. Figure 5 shows an example where a user has highlighted electrode G27, as well as a functional map in the category of *“motor”*. The information regarding the seizure type of an electrode is displayed, and the note for the selected functional map. Since G27 has no seizure type, no caption is displayed for seizure type or interictal population. New connections will be drawn if the user selects an alternative functional map. The user can also press the *“Show All Tags”* button to display the caption of each electrode, as seen in Figure 6. Finally, Figure 7 shows the complete finished rendering of three subjects.

We added WebGL’s dat. GUI for direct visibility and to enable interactive manipulation for the user. We separate the electrode and functional map data from the slice and mesh controllers by using two panels. Figure 4 shows the one panel in two segments responsible for controlling the slices and hemisphere meshes. Users can use this panel to select a specific slice number, control the opacity of the slices and hemisphere, and toggle visibility of the slices. This makes it easier to find needed information more quickly.

### 4.4 Setup, Installation, and Deployment

N-Tools-Browser is designed to run client-side only and can be used with any web server. The installation and setup of N-Tools-Browser involve specific steps associated with the overall workflow established in Figure 2. A user first runs the N-Tools-Elec pipeline on a local workstation, generating a brain surface file (reconstructed from an input T1-weighted MRI *via* Freesurfer) and a JSON file containing electrode information (see section Electrode Visualization on 3D brain surface). All three, the T1 MRI, Freesurfer mesh file, and JSON file, are loaded into then uploaded to the patient’s directory on a webserver. For web security and health information protection, the T1 MRI is defaced, and the patient’s directory contains no personal information other than the patient ID. In the case of NYU, the webserver is also protected by campus firewall, accessible to authorized users only. The user should browse to the Github repository for N-Tools-Browser, which can be found at https://github.com/ntoolsbrowser/ntoolsbrowser.github.io/, then make a copy of the source code by forking the repository.

{

“subjID”:“fsMNI”,

“totalSeizType”:2, “SeizDisplay”[“SeizureType3″,“SeizureType4″,“intPopulation”,“funMapping”], 

“elecID” [“G01″,“G02″,“G03″,“G04″,“G05″,“G06″,“G07″,“G08″,“G09″, ..., “G64”],

“coorX” [−37.5, −47.5, −55.83333, −59.3, −62.5, −65.3, −65.5, −65, −40.5, ..., −59.9],

“coorY” [−41.5, −41, −39.16667, −38.9, −37.16667, −35.3,−34.5, −33,−31.5, ..., 41.5],

“coorZ” [47.5, 38, 26.83333, 16.3, 2.83333, −8.1, −19.5, −33, 49.5, 38.83333, ..., −22.5],

“elecType” [“G″,“G″,“G″,“G″,“G″,“G″,“G″,“G″,“G″, ..., “G”],

“intPopulation” [2,2,0,0,0,0,6,0,0, ..., 0],

...

“fmapMotor”[”“,”“,”“,“Right facial (chin, lip)deviation”,”“,”“, ...,””],

...

}

Listing 1 A small segment of what the generated JSON file looks like. The “...” in the arrays indicate additional values not shown, as the arrays in the actual JSON can contain hundreds of elements.

A core intermediate step in the workflow is generating a label map containing patients’ electrode locations mapped onto 2D brain slices. This step is achieved via a Python script, which takes the electrode’s location in 3D, calculates the 2D position, and maps the electrodes onto brain slices in the coronal, sagittal, and axial positions. This step must be completed before visualization in N-Tools-Browser. N-Tools-Browser also retrieves its inputs from the same directory. Therefore, we set the script to output the label map to the patient’s subject folder located in the parent directory stored the script on the webserver. To run the script, a user would first navigate to the umb_ntools directory where the Python script, electrode_slice.py, is located using the cd command. The user would then run the script by providing the following three arguments in the command-line: 1) SubjectID, 2) Path to subject’s.json file, and 3) Path to subject’s T1. nii file. Both pathnames can either be an absolute pathname or a relative pathname.

$ cd p r e p r o c e s i n g.

$./electrode_slice.py fsMNI .. /fsMNI/fsMNI.json .. /fsMNI/fsMNI_T1.nii

Listing 2 An example of running the script for subject ID *“fsMNI.”*

After running the Python script, depending on the subject’s seizure types, one or more NIfTI files are generated in the subject’s directory. By default, if the seizure type is labeled “funMapping,” then a file with the name *subject_default_labels.nii* would be saved. For all other seizure types, a file with the name *subject_seizType_labels.nii* would be saved. Following the label map generation, a user at NYU could then proceed to N-Tools-Browser, select NYU mode, and enter the subject ID to load all the relevant files for visualization.

A demo of N-Tools can be found at the following link: https://ntoolsbrowser.github.io/. Two sample datasets are available for the demo: *fsaverage* and *fsMNI*. Users can enter either dataset into the subject_ID box, select UMB mode, and click on *“Load Data!”* to load the sample files. Although N-Tools currently only supports structural MRI data at this time, the open source nature of the tool allows future support of additional formats and modalities.

## 5 Evaluation

Based on our design objectives defined in Section 3, we evaluated the usability of the user interface with two domain experts. We selected two expert clinicians from the NYU School of Medicine who are familiar with working with various software tools for epilepsy research. Expert one is a 46-year-old male, and expert two is a 45-year-old female, both of whom are experienced neuroscientists performing tasks involving the extraction or analysis of data from visualization. From the design objectives, we derived a structured list of tasks for our experts to evaluate our tool, which is described in Section 5.1.

Besides the tasks, we additionally used the standard NASA-TLX survey (Hart and Staveland, 1988) to assess the workload with tasks concerning mental demand, frustration, etc. We also asked some general usability questions on how the users perceived the overall usability and some open-ended questions to collect user comments as qualitative feedback.

### 5.1 Tasks

After the introduction, we asked our domain experts to perform the following list of tasks during the user study in two rounds with two different subjects (full instructions are provided as Supplemental Material):

Task 1 Search and load subject NY704 (training) and subject NY836.

Task 2 Identify which electrodes are connected in the functional mapping group named “motor”.

Task 3 Find the coordinates of electrode *“G56”* (training) and *“G008”*, and identify the anatomical region.

Our tasks were structured with increasing difficulty. Same as in a real-world scenario, Task one had the goal to search and load specific data in which a domain scientist might be interested. Next, Task two was concerned with identifying the functional mapping. Task three was particularly for experienced neuroscientists, finding an anatomical region.

### 5.2 User Study and Results

We performed our user study virtually in separate Zoom meetings with our domain experts. The meetings took around 15 min per expert. We began by asking the experts to open the tool in the web browser and explaining the main functionalities. Then, we gave them 5 min to explore the tool to become familiar with its functionality and ask any questions. Once this short period was complete, we performed two rounds of three tasks.

Task one had the quickest completion time since it simply involved searching and loading the different subjects. The subject used after the training round contained significantly more data than the first, which accounts for the time difference for the two rounds. We observed that Task two took most of the time since the experts occasionally had to change camera angles or make particular 3D objects invisible to obtain a better view. Both experts had a much easier time with Task 3, which involved finding an electrode and identifying its anatomical region. They quickly found this information, such as the electrode coordinates, by selecting an electrode from the drop-down menu and then using their anatomical knowledge to identify the appropriate anatomical region A task summary along with completion times is shown in Table 1.

**TABLE 1:** We measured how fast two experts can perform common visual exploration tasks with N-Tools-Browser. After a 5 min period of voice-guided training, the experts were asked to perform the tasks on another previously unseen dataset without help. Both experts were able to successfully complete the tasks.

Task	Expert 1 Time [s]	Expert 2 Time [s]	Average Total **[s]**
Task 1 (Search and Load), *training*	3	5	4
Task 2 (Identify Functional Mapping), *training*	88	25	56.5
Task 3 (Find Anatomical Region), *training*	10	36	23
Task 1 (Search and Load)	13	10	11.5
Task 2 (Identify Functional Mapping)	43	60	51.5
Task 3 (Find Anatomical Region)	13	20	16.5

Our supplemental questionnaire had our experts rate their experience with different features on a Likert scale from one to 7. The 7-point ordinal scale allows the respondents to rate the degree to which they agree or disagree with a statement with one being *“totally disagree”* and seven being *“totally agree.“*. Overall, we found that their responses indicated that they found it easy to fulfill their tasks and were confident with having the correct results, both experts rating either a six or 7 (out of 7) for each. They also strongly or totally agreed that both the 2D and 3D visualizations were helpful and understandable.

The qualitative feedback from the experts was positive, mentioning *“we eagerly anticipate using this program!“*, *“this is very cool”*, and *“I like the tool.“*. One expert, when commenting on the functional mapping visualization and captioning, said *“I really like that you can add the actual findings.“*. The user also liked the hovering option on the electrodes to show the motor functions that are then visible in the labels. They also commented on possible additional features or improvements. For example, since both experts started Task two and Task three by making the rendered 3D slices invisible, they suggested that this should be the default setting after loading. Other suggestions included *“a cursor or other marker should identify the selected electrode in the 2D slices,”* and using less significant digits when displaying the 3D coordinates of an electrode.

**FIGURE 8:**
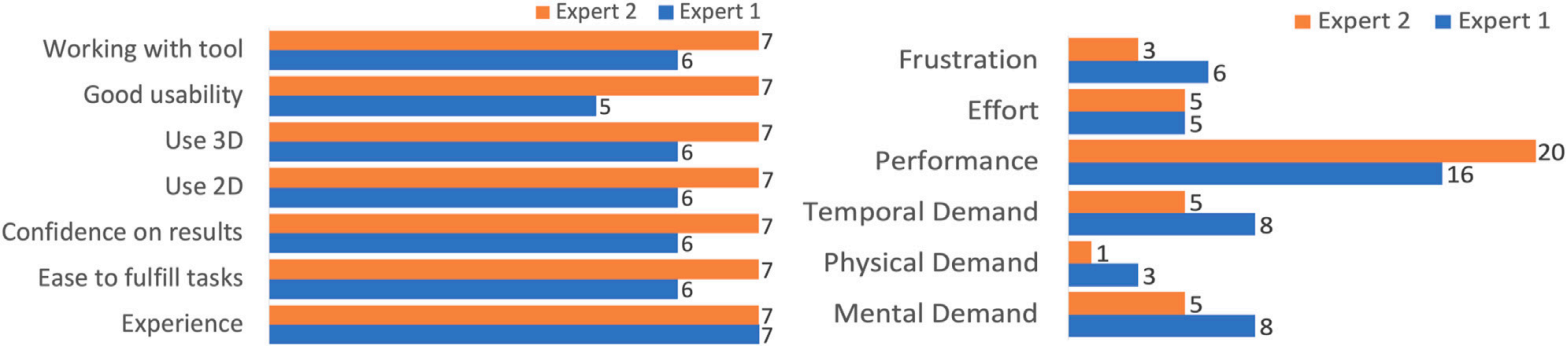
Left: We collected the qualitative feedback from neuroscientists after evaluating the N-Tool-Browser. The experts gave positive feedback on the scale range from one to seven, and were satisfied with their results. Right: We also performed the standardized NASA-TLX questionnaire and the experts reported low frustration and high performance on the scale range from 1–21.

The NASA TLX workload assessment showed that experts found it overall easy to fulfill the tasks. The scale is ranked from 0 to 21 where 0 depicts a lower perceived workload, and 21 a higher perceived workload. Both experts rated the mental, physical, and temporal demand as low, with the highest being a report of eight out of the 21-point scale for cognitive demand. When assessing the performance of the tool, the question *“How successful were you in accomplishing what you were asked to do?”* was asked, and both experts responded in the high to very high range, with scores of 16 and 20 (out of 21) The complete feedback from both questionnaires can be seen in Figure 8.

## 6 Conclusion and Outlook

We present N-Tools-Browser as an extension of the pre-existing N-Tools-Elec ECoG toolkit. As web-based medical imaging software, N-Tools-Browser allows neuroscientists to explore the toolkit output without any additional software installation. Our application also allows quick access to electrodes’ positions, functional units, and anatomical information.

Being a prototype, N-Tools-Browser also has some limitations. First, the pre-processing pipeline that generates input data for N-Tools-Browser contains both MATLAB and Python scripts (see Section 4.4). These extra required pre-processing steps may present challenges to clinicians who may not be experienced with the how to run the MATLAB pipeline or Pythons script to generate the necessary outputs As Python packages for ECoG data processing are emerging (Combrisson et al. (2019); Gramfort et al. (2013); Murugesan et al. (2017)), we believe that a pure Python pipeline would be easier to maintain and update. Second, while the input NIfTI and Freesurfer surface files are in a standard format, the format of the JSON electrode profile is specific to the installation at NYU CEC. Therefore, data reformatting may be required to use this toolkit at other centers. Future work involves implementing the standardized BIDS format (Gorgolewski et al., 2016) to interface other neuroimaging tools with N-Tools-Browser as well as converting the custom MATLAB pipeline entirely to Python code which runs directly in N-Tools-Browser as the data is loaded. N-Tools-Browser is not certified for clinical use and should be used for research purposes only.

## Data Availability

Publicly available datasets were analyzed in this study. This data can be found here: fsaverage and fMNI datasets used for training only https://github.com/ntoolsbrowser/ntoolsbrowser.github.io/tree/main/data.
