# Supplementary material for: N-Tools-Browser: Web-Based Visualization of Electrocorticography Data for Epilepsy Surgery
Source: Front Bioinform. 2022 Apr 21;2:857577. doi: 10.3389/fbinf.2022.857577 (PMC9580919; doi:10.3389/fbinf.2022.857577)
Supplement: Supplementary file 1 [file DataSheet2.PDF]

Task List

Please complete the following tasks to the best of your ability.

Task 1. Loading Data

From the search page, please load a subject in “NYU” mode.

- Choose “NY704” for the first round.
- Choose “NY836” for the second round.

Task 2. Functional Mapping

Identify which electrodes are connected in the functional mapping group named “motor.”

Task 3. Locating Electrodes

For the subject “NY704”, use electrode “G56.”
For the subject “NY836”, choose electrode “G008.”

a. Identify the x,y,z coordinates of the electrode specified for your sample.
b. Identify the anatomical region where this electrode resides.

Thank you again for your participation! Please click here to fill out a survey regarding your experience.
