# Supplementary material for: N-Tools-Browser: Web-Based Visualization of Electrocorticography Data for Epilepsy Surgery
Source: Front Bioinform. 2022 Apr 21;2:857577. doi: 10.3389/fbinf.2022.857577 (PMC9580919; doi:10.3389/fbinf.2022.857577)
Supplement: Supplementary file 2 [file DataSheet3.PDF]

Questionnaire

Demographic data and questions to the workload

* Required

1. Your name *

____

2. Age *

____

3. Gender *

Mark only one oval.

- ( ) Female
- ( ) Male
- ( ) Prefer not to say
- ( ) Other: _______________

4. Estimate your experience level regarding neuroscience *

Mark only one oval.

	1	2	3	4	5	6	7	
weak	( )	( )	( )	( )	( )	( )	( )	strong

5. Did you ever perform tasks involving the extraction or analysis of data from visualizations? *

Mark only one oval.

- ( ) Yes
- ( ) No

6. In general, I found it easy to fulfill the tasks. *

Mark only one oval.

	1	2	3	4	5	6	7	
Totally Disagree	( )	( )	( )	( )	( )	( )	( )	Totally Agree

7. I am confident that my results are correct. *

Mark only one oval.

	1	2	3	4	5	6	7	
Totally Disagree	( )	( )	( )	( )	( )	( )	( )	Totally Agree

8. The 2D visualizations / 2D Slices were understandable and helpful. *

Mark only one oval.

	1	2	3	4	5	6	7	
Totally Disagree	( )	( )	( )	( )	( )	( )	( )	Totally Agree

9. The visualization of the 3D electrodes were very pleasing. *

Mark only one oval.

	1	2	3	4	5	6	7	
Totally Disagree	⬭	⬭	⬭	⬭	⬭	⬭	⬭	Totally Agree

10. The usability was very good. *

Mark only one oval.

	1	2	3	4	5	6	7	
Totally Disagree	⬭	⬭	⬭	⬭	⬭	⬭	⬭	Totally Agree

11. I liked working with this tool. *

Mark only one oval.

	1	2	3	4	5	6	7	
Totally Disagree	⬭	⬭	⬭	⬭	⬭	⬭	⬭	Totally Agree

NASA Task Load Index

Hart and Staveland's NASA Task Load Index (TLX) method assesses work load on five 7-point scales. Increments of high, medium and low estimates for each point result in 21 gradations on the scales.

This is an image of the Task load questionnaire. We added the questions below so that you can fill them out by inserting a number on the scale.

NASA Task Load Index

Hart and Staveland's NASA Task Load Index (TLX) method assesses work load on five 7-point scales. Increments of high, medium and low estimates for each point result in 21 gradations on the scales.

Name	Task	Date

Mental Demand — How mentally demanding was the task?

Very Low — Very High

Physical Demand — How physically demanding was the task?

Very Low — Very High

Temporal Demand — How hurried or rushed was the pace of the task?

Very Low — Very High

Performance — How successful were you in accomplishing what you were asked to do?

Perfect — Failure

Effort — How hard did you have to work to accomplish your level of performance?

Very Low — Very High

Frustration — How insecure, discouraged, irritated, stressed, and annoyed wereyou?

Very Low — Very High

12. Mental Demand: From a scale from 0 (very low) to 21 (very high) : How mentally demanding was the task? *

13. Physical Demand: From a scale from 0 to 21 where 0 is very low and 21 is very high: How physically demanding was the task? *

14. Temporal Demand: From a scale from 0 (very low) to 21 (very high): How hurried or rushed was the pace of the task? *

15. Performance: From a scale from 0 (Failure) to 21 (Perfect): How successful were you in accomplishing what you were asked to do? *

16. Effort: From a scale from 0 (very low) to 21 (very high): How hard did you have to work to accomplish your level of performance? *

17. Frustration: From a scale from 0 (very low) to 21 (very high): How insecure, discouraged, irritated, stressed, and annoyed were you? *

18. Any other/additional feedback

This content is neither created nor endorsed by Google.

Google Forms
