# Supplementary material for: N-Tools-Browser: Web-Based Visualization of Electrocorticography Data for Epilepsy Surgery
Source: Front Bioinform. 2022 Apr 21;2:857577. doi: 10.3389/fbinf.2022.857577 (PMC9580919; doi:10.3389/fbinf.2022.857577)
Supplement: Supplementary file 3 [file DataSheet1.PDF]

N-Tools-Browser User Study

If not already provided, the initial search page for the demo can be found by clicking here.

Thank you for participating in our expert study of N-Tools-Browser! Your feedback is much appreciated. Our goal today is to have you complete two rounds of three tasks which will help us assess the usability of this application. Afterwards, please follow the link at the bottom of the task-list document to take a survey regarding your experience.

There will be a five minute period where you can ask any questions, however, you may start whenever you are ready. After this period, the study will begin.

When you are ready, please click the link below to receive your list of tasks.

Expert Study Task List
